# Prospect of Stem Cell Therapy and Regenerative Medicine in Osteoporosis

**DOI:** 10.3389/fendo.2020.00430

**Published:** 2020-07-03

**Authors:** Babak Arjmand, Masoumeh Sarvari, Sepideh Alavi-Moghadam, Moloud Payab, Parisa Goodarzi, Kambiz Gilany, Neda Mehrdad, Bagher Larijani

**Affiliations:** ^1^Cell Therapy and Regenerative Medicine Research Center, Endocrinology and Metabolism Molecular-Cellular Sciences Institute, Tehran University of Medical Sciences, Tehran, Iran; ^2^Metabolomics and Genomics Research Center, Endocrinology and Metabolism Molecular-Cellular Sciences Institute, Tehran University of Medical Sciences, Tehran, Iran; ^3^Obesity and Eating Habits Research Center, Endocrinology and Metabolism Molecular-Cellular Sciences Institute, Tehran University of Medical Sciences, Tehran, Iran; ^4^Brain and Spinal Cord Injury Research Center, Neuroscience Institute, Tehran University of Medical Sciences, Tehran, Iran; ^5^Department of Biomedical Sciences, University of Antwerp, Antwerp, Belgium; ^6^Integrative Oncology Department, Breast Cancer Research Center, Motamed Cancer Institute, Academic Center for Education, Culture and Research (ACER), Tehran, Iran; ^7^Reproductive Immunology Research Center, Avicenna Research Institute, Academic Center for Education, Culture and Research (ACER), Tehran, Iran; ^8^Elderly Health Research Center, Endocrinology and Metabolism Population Sciences Institute, Tehran University of Medical Sciences, Tehran, Iran; ^9^Endocrinology and Metabolism Research Center, Endocrinology and Metabolism Clinical Sciences Institute, Tehran University of Medical Sciences, Tehran, Iran

**Keywords:** cell therapy, chronic diseases, mesenchymal stem cells, osteoporosis, regenerative medicine

## Abstract

The field of cell therapy and regenerative medicine can hold the promise of restoring normal tissues structure and function. Additionally, the main targets of stem cell-based therapies are chronic diseases and lifelong disabilities without definite cures such as osteoporosis. Osteoporosis as one of the important causes of morbidity in older men and post-menopausal women is characterized by reduced bone quantity or skeletal tissue atrophy that leads to an increased risk of osteoporotic fractures. The common therapeutic methods for osteoporosis only can prevent the loss of bone mass and recover the bone partially. Nevertheless, stem cell-based therapy is considered as a new approach to regenerate the bone tissue. Herein, mesenchymal stem cells as pivotal candidates for regenerative medicine purposes especially bone regeneration are the most common type of cells with anti-inflammatory, immune-privileged potential, and less ethical concerns than other types of stem cells which are investigated in osteoporosis. Based on several findings, the mesenchymal stem cells effectiveness near to a great extent depends on their secretory function. Indeed, they can be involved in the establishment of normal bone remodeling via initiation of specific molecular signaling pathways. Accordingly, the aim herein was to review the effects of stem cell-based therapies in osteoporosis.

## Introduction

Osteoporosis as a chronic and long-term skeletal disorder is more common in senile people (in men after age 65 and women after age 55 years) (1–4). Accordingly, it is responsible for most of the elderly fractures through decreasing the bone mass and mineral density (BMD) (1, 5, 6). Moreover, it has been reported that osteoporosis occurs when there is an imbalance between bone cells function (7, 8). In 1993, osteoporosis is defined as “progressive systemic skeletal disease characterized by low bone mass and microarchitectural deterioration of bone tissue, with a consequent increase in bone fragility and susceptibility to fracture” by WHO (9–12). The proximal ends of the humerus and femur, the distal end of the radius, and the vertebral column are more susceptible to the osteoporotic fractures in contrast to other parts of the bone (13–15). Additionally, the hip fracture can be considered as the serious complication with high morbidity and mortality (15–17). Given the fact that the life expectancy universally is increasing and subsequently osteoporosis becomes a growing global problem with a great impact on quality of life, selecting powerful approaches for disease managing is essential. In this respect, there is no practical pharmaceutical cure (18). Recently, stem cell therapies have attained remarkable clinical consideration with a promising strategy for regenerative medicine and tissue engineering to treat various types of diseases including osteoporosis (19–26). Herein, discuss the effects of stem cell-based therapies in osteoporosis is the main objective of this review.

## Bone Biology; Signaling Pathways; Bone Modeling and Remodeling

**Figure 1:**
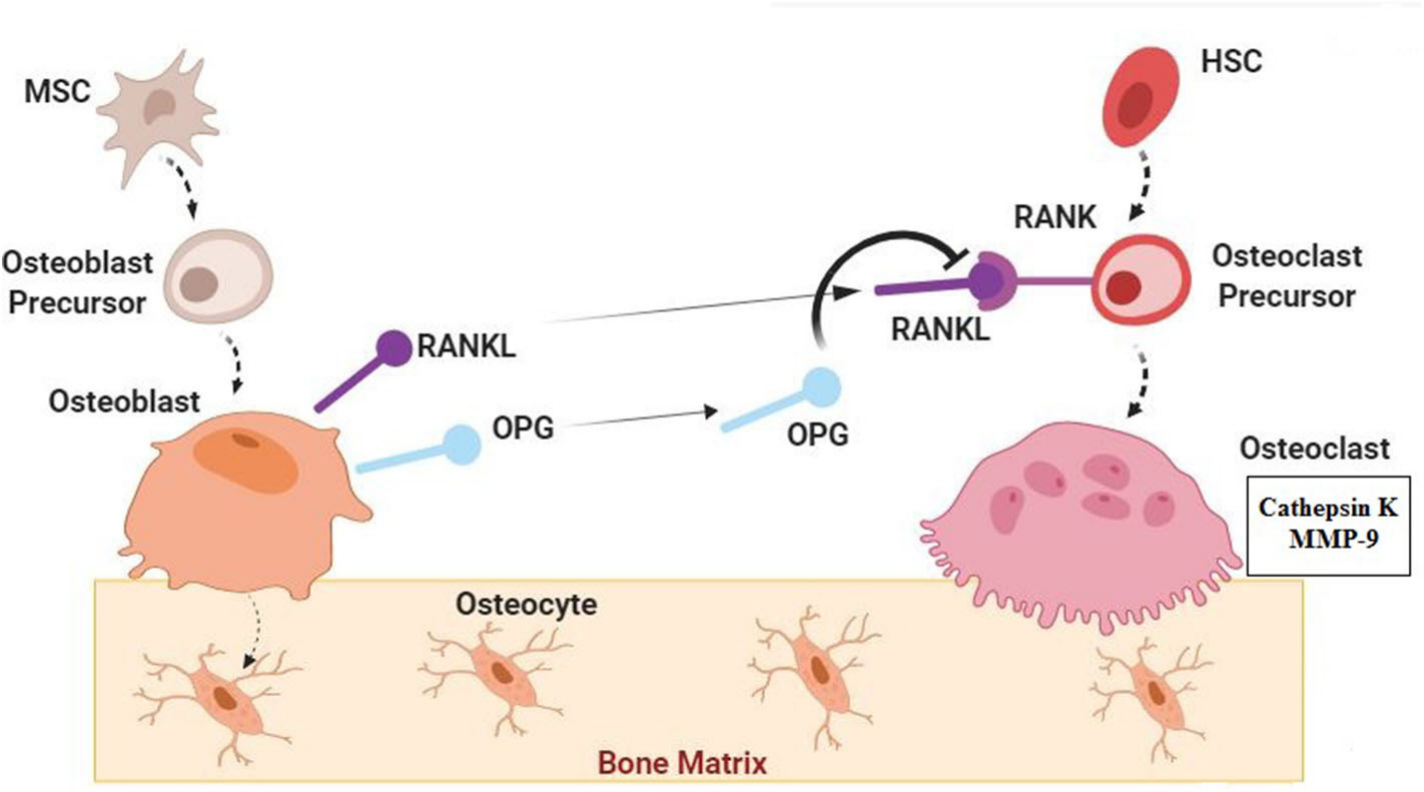
Normal Bone Biology; Signaling Pathways. Bone as a dynamic tissue undergoes modeling and remodeling by activation of osteoblasts, osteoclast, and osteocytes. Mesenchymal stem cells (MSCs) are proliferated and differentiated into osteoblasts. Some signaling molecules have important roles in osteoblast turnover and function including runt-related transcription factor 2 (Runx2), osterix (Osx), ß-Catenin, activating transcription factor 4(Atf4), activator protein 1(AP-1) family, fibroblast growth factors (FGFs), transforming growth factor β (TGF β), insulin-like growth factor 1 (IGF-1), bone morphogenetic proteins (BMPs), Notch, Wnt, and parathyroid hormone (PTH) (27–33). Osteoblasts which are trapped in the bone matrix are called osteocytes. Osteoclasts are derived from the hematopoietic stem cells (HSC) through the stimulation by receptor activation of NF-κB ligand (RANKL) from osteoblasts. Osteoprotegerin (OPG) which is also secreted by osteoblasts can interfere with the RANKL and inhibit osteoclastogenesis. Osteoclasts can secrete cathepsin K and matrix metallopeptidase 9 (MMP-9) in extracellular space. Some inflammatory cytokines such as. interleukin1 (IL-1), interlukin-6 (IL-6), and tumor necrosis factor-α (TNFα) can be involved in osteoclast differentiation and function (34–37). In normal condition Bone formation (by osteoblasts) and resorption (by osteoclasts) are in balanced for bone mass maintenance (34, 38, 39).

Bone as a highly dynamic tissue continuously undergoes modeling and remodeling via activation of bone cells (osteoblasts, osteoclast, and osteocytes) (Figure 1) (40–42). Herein, modeling is defined as separately happening of bone formation and resorption on the bone surface and remodeling is known as the coupling between bone formation and resorption for regeneration (43–46). The process of developing new bone material by osteoblasts is called bone formation (ossification or osteogenesis) which commences about 6 weeks after fertilization in embryos. There are two types of bone formation, including intramembranous and endochondral (27, 47). During intramembranous bone formation, mesenchymal stem cells (MSCs) are proliferated and differentiated into osteoblasts in areas of embryonic connective tissue which contain high vascularization. Additionally, the intramembranous bone formation that is involved in the formation of the flat bones of the clavicles, skull, and the mandible is known as a procedure of bone formation from fibrous membranes (48, 49). The endochondral bone formation is befallen at three sites including the physis, the epiphysis, and the cuboidal bones of the carpus and tarsus. It is a procedure in which the cartilage is commonly replaced by bone for the formation of the growing skeleton (50–52). In general, bone formation is controlled by various growth factors, cytokines, and hormones (40, 53, 54). Therein, osteoblasts can reply to these external signals through various signaling pathways and control the specific gene expression for cell fate determining (28, 29, 55). Accordingly, there are some signaling molecules with critical roles in osteoblast turnover including runt-related transcription factor 2 (Runx2), osterix (Osx), ß-Catenin, activating transcription factor 4 (Atf4), and activator protein 1(AP-1) family. Indeed, they have momentous roles in osteoblast differentiation and osteoblastogenesis to promote bone formation (27–33). Moreover, it has been demonstrated that fibroblast growth factors (FGFs), transforming growth factor β (TGF β), insulin-like growth factor 1 (IGF-1), bone morphogenetic proteins (BMPs), Notch, Wnt, and parathyroid hormone (PTH) have effective roles in the bone formation process (56–59). Bone formation and resorption must be balanced for bone mass maintenance (34, 38, 39). Bone resorption is the process of minerals dissolution and organic matrix degradation by osteoclasts, which depends on the osteoclasts secretions into the extracellular space (60–63). Some more important types of osteoclasts secretions are lysosomal enzymes (e.g., cathepsin K) and matrix metallopeptidase 9 (MMP-9) (41, 64, 65). Osteoclasts arise from the hematopoietic stem cells (HSC) via stimulation of receptor activation of NF-κB ligand (RANKL) and the monocyte/macrophage colony-stimulating factor (M-CSF) from osteoblasts membrane surface (60, 66, 67). RANKL and M-CSF are interacted with their receptors present on osteoclast precursors to stimulate osteoclast proliferation and differentiation (60, 68, 69). However, there is another signaling molecule called osteoprotegerin (OPG) which is also secreted by osteoblasts to interfere with the RANKL for inhibition of osteoclastogenesis (70–73). According to investigations, some inflammatory cytokines e.g., interleukin1 (IL-1), interlukin-6 (IL-6), and tumor necrosis factor-α (TNFα) can be involved in osteoclast differentiation and function (34–37). Several findings have indicated that imbalance between osteoclasts and osteoblasts functions can lead to some skeletal disorders including osteoporosis. In fact, these disorders are the consequence of decreased in osteoblast activity and/or increased in osteoclast activity (8, 41).

## An Overview on Osteoporosis: Imbalance Between Bone Formation and Resorption

As a result of the aging process, reduction in osteoblast number, function, and longevity, lead to bone formation decreasing However, bone resorption is exceeded due to sex hormones defection. Accordingly, individuals are predisposed to osteoporosis and osteoporotic bone fractures (74–77). In fact, osteoporotic bones due to low bone mass are fragile and brittle. The compression fractures of the vertebrae and traumatic fractures of the femoral neck and the wrist are the main issues of osteoporosis. Nevertheless, the hip fractures due to their burden are more considerable and need more attention. It is estimated that by 2050 the number of hip fractures will be more than 6 million and almost the 75% of them will be occurred in the developing countries (9). Osteoporosis can be followed by various complications and disorders. Usually, low levels of estrogen in post-menopausal women is the most well-known factor (78). In clinical diagnostic techniques of osteoporosis, dual x-ray absorptiometry [DXA] is approved as a gold standard approach to diagnose and follow the osteoporosis by calculating BMD (79). The WHO defines a set of categories to diagnose osteopenia and osteoporosis. These guidelines are based on T-score and Z-score. T-score shows the number of standard deviations above or below the mean reference value for 30 year-old healthy adults. However, Z-score measures the BMD regards to the average BMD of the same age and gender (80). According to the guidelines, a score above −1 is considered normal, a score between −1 and −2.5 indicates osteopenia, and a score below −2.5 portends the osteoporosis (79). Hereupon, for individuals with osteoporosis diagnosed, various treatments are recommended to increase the quality of life and decrease the economic burden on health care system (1).

## Current Treatments and Limitations

Osteoporosis cannot be cured but some of the pharmacological and non-pharmacological treatment approaches can manage it (Table 1) through the strengthening the bones and preventing the consequent fractures. In this context, using bisphosphonates, selective estrogen receptor modulators (SERMs), teriparatide, denosumab, calcitonin, and hormone replacement therapy (HRT) are the approved methods as the pharmacological treatments for osteoporosis (94). Additionally, some of the non-pharmacological treatments are including nutritional therapy, physical exercises, vertebroplasty, and kyphoplasty. Despite the preventive and therapeutical effects of these treatments, there are some limitations and side effects around using them. Hence, it is needed to apply new and more effective approaches with fewer side effects for osteoporosis management.

**Table 1:** Some of the pharmacological and non-pharmacological treatments for osteoporosis (81–93).

**Treatment**	**Positive effects**	**Side effects/limitations**	**Type of treatment**
Bisphosphonates	- Can decrease both hip and spine fracture risk through maintaining the bone mineral density	- Osteonecrosis of jaw - Gastrointestinal and renal discomfort - Atypical femoral fractures - Acute influenza-like illness	Pharmacological
Teriparatide	- As a recombinant parathyroid hormone can be used to stimulate osteoblasts to reconstruct the osteoporotic bone - Can improve the bone mineral density and the bone architecture - Considered as an impressive agent to decrease the vertebral, non-vertebral, and hip fracture risks	- Inflammation of the nose - Diarrhea - Constipation - Joint Pain	Pharmacological
Hormone replacement therapy	- Safe and cost-benefit approach with positive effects on preventing the vertebral and non-vertebral fractures	- Cardiovascular, thromboembolic, and gallbladder discomforts, breast and endometrial cancers	Pharmacological
Selective estrogen receptor modulators	- Can be a good choice to prevent the number of hormone replacement therapy related complications - Can improve the bone mass and reduce the fracture risk	- Have some limitations in preventing non-vertebral fractures and also have extra-skeletal side effects	Pharmacological
Physical exercises	- Can lead to bone loss reduction - Can conserve remain bone tissue - Can reduce the risk of bone fractures caused by falls	- Some types of physical exercises such as abdominal sit-ups or loaded forward flexion of the spine can increase the risk of the spine compression fractures.	Non-pharmacological
Vertebroplasty	- Can relieve symptoms associated with vertebral compression fractures	- May lead to spinal cord or nerve root injury - May lead to infection - May lead to pulmonary embolus	Non-pharmacological
Kyphoplasty	- Can relieve symptoms associated with vertebral compression fractures	- May lead to cement leaks - May lead to infection - May occur balloon rupture	Non-pharmacological

## Cell Therapy as a Novel Approach

The clinical demand for new therapeutic methods has been led to progress in stem cell therapy and regenerative medicine (23, 95). In other words, stem cell-based therapies are becoming increasingly important in treatment of chronic and long-lasting diseases (96, 97). However, there are several parameters which need to be optimized for maximizing stem cell-based therapies potential. In this context, various basic and clinical studies related to the effects of stem cell-based therapies on diseases with no definite treatments were performed (22, 98, 99). Accordingly, some investigations were also conducted in the field of stem cell therapy for osteoporosis. Herein, the application of different types of stem cells including embryonic, induced pluripotent, and MSCs along with their secretion factors were evaluated to treat osteoporosis (100–102).

## Mechanism of Stem Cells Function in Bone Remodeling and Osteoporosis

Osteoporosis is a multifactorial disorder with endogenous and exogenous components (103, 104). Cell-based regenerative medicine can be invaluable in osteoporosis treatment through bone resorption modulation, fractures susceptibility reduction, and lost mineral density enhancement. These are possible by increasing the number of progenitor stem cells and improve the function of stem cells (proliferation and differentiation into bone-forming cells) (20, 102, 105, 106). Since the bone tissue repair cascade can be controlled by local signals from various cytokines and growth factors through the inducing osteoprogenitor cells migration, differentiation, proliferation, revascularization, and extracellular matrix production (56, 107, 108), stem cells (especially MSCs) can support bone regeneration by secreting bioactive molecules such as IGF-1, TGF-β, vascular endothelial growth factor (VEGF), angiogenin, hepatocyte growth factor (HGF), IL-6, and etc. (56, 109–113). On the other hand, MSCs derived exosomes are other factors which their effects on preventing the bone loss and promoting bone remodeling processes (during osteogenesis, osteoclastogenesis, and angiogenesis) have been demonstrated *in vitro* and *in vivo* (114–116).

## Embryonic, Induced Pluripotent, and Embryonic Like Stem Cells in Osteoporosis

Although particular protocols are demanded to direct differentiation of embryonic stem cells (ESCs) (from the inner cell mass of a blastocyst) and induced pluripotent stem cells (iPSCs) (embryonic–like stem cells reprogrammed from adult cells) toward the osteoblasts and osteocyte-like cells (bone-forming cells), some of investigations were shown that application of these most known pluripotent stem cells in osteoporosis treatment is limited due to ethical concerns (20, 117, 118). Recently, implementation of very small embryonic-like stem cells (VSELs) (non-hematopoietic pluripotent cells that express embryonic characteristics markers and stored during the organogenesis in organs and tissues) as the autologous treatment for decreasing the aging processes which lead to osteoporosis and other skeletal disorders is taken into consideration. However, according to some studies, VSELs population will decrease with aging (20, 119, 120).

## Mesenchymal Stem Cells in Osteoporosis

**Figure 2:**
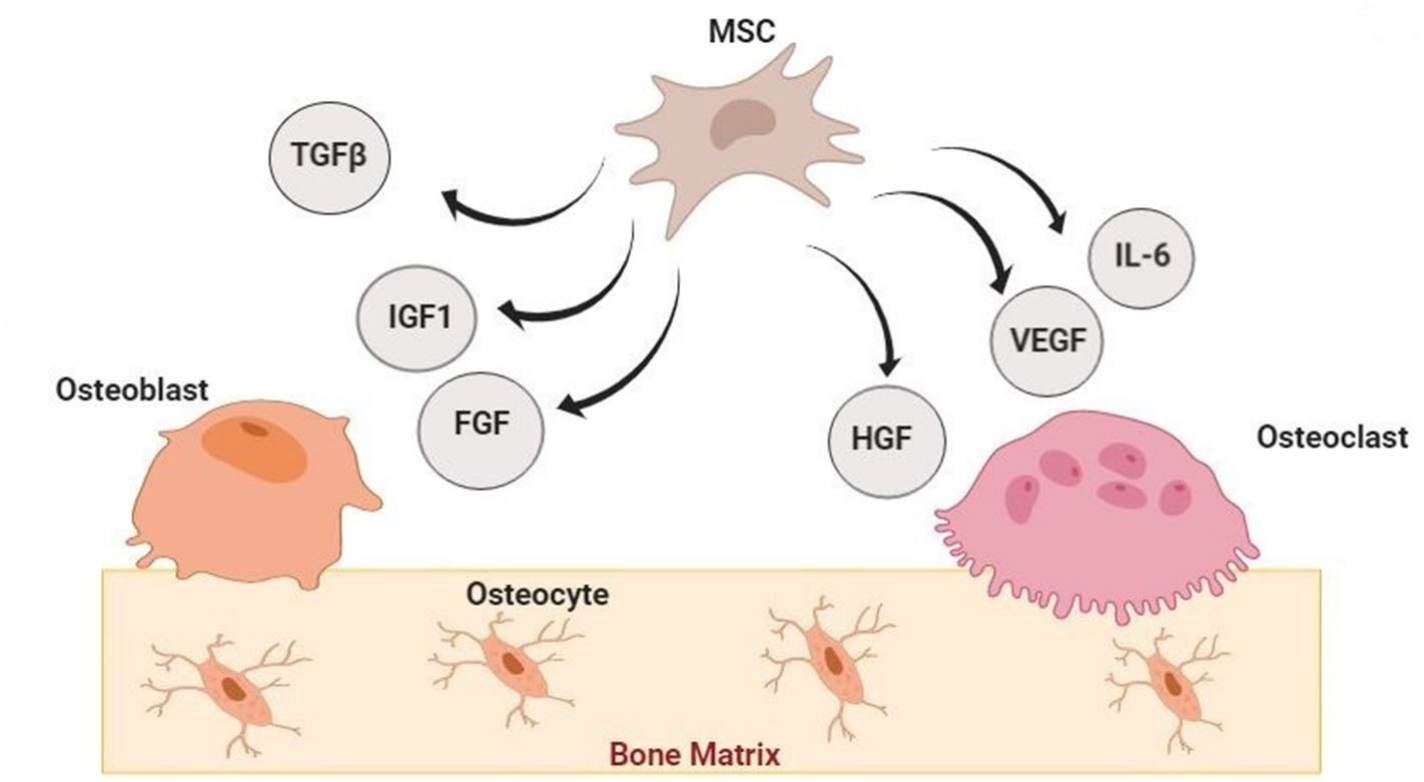
Paracrine Effects of Mesenchymal Stem Cells in Bone Regeneration. Mesenchymal stem cells (MSCs) can participate in bone regeneration by secreting bioactive molecules such as Insulin-like growth factor 1 (IGF-1), Transforming growth factor β (TGF-β), vascular endothelial growth factor (VEGF), hepatocyte growth factor (HGF), interleukin−6 (IL-6), and fibroblast growth factor (FGF) (140–143).

In osteoporosis, there is a reduction in endogenous MSCs function (proliferation, differentiation, and consequently bones formation). Accordingly, they are the most common types of stem cells investigated in osteoporosis treatment. In this respect, examples of MSCs transplantation in osteoporotic animal models and humans were shown in Table 2. MSCs are an important example of non-hematopoietic stem cells with less ethical concerns and numerous advantages for clinical usage, containing accessibility and ease of harvesting, immunosuppressive outcomes, multi-lineal differentiation ability (especially ability to differentiate into osteoblasts), and any possibility of malignant transformation (21, 131–133). Additionally, as a subset of stromal stem cells, they can be obtained from various tissue sources. Bone marrow derive MSCs (BM-MSCs) with high osteogenic differentiation capability are the most common types of MSCs which have been used for osteoporosis (20, 24, 134–136). Herein, accumulating evidence indicates that alternation in the molecular mechanisms which modulate osteoblast differentiation in MSCs will make the MSC therapies reliable and more effective for osteoporosis (105, 137–139). While in accordance with other studies the most therapeutic impressions of MSCs are due to their supporting regenerative microenvironment ability and paracrine effects rather than their differentiation ability. In other words, MSC transplantation might open a new chapter in osteoporosis treatment specifically through paracrine effects (Figure 2) (140–143).

**Table 2:** Examples of MSCs transplantation in osteoporotic animal models and humans.

**UC-MSC**	**ADMSC**	**BM-MSC**	**Stem cell type**
−30 ovariectomized rats (2018) (121) - 30 Wistar rats (2018) (122) - 20 e Balb/c nude mice (2008) (123)	−30 ovariectomized rats (2018) (124) - 22 SAMP6 mice (2014) (125) - 27 Balb/c nude mice (2011) (126)	−60 estrogen deficiency-induced osteoporotic - C57/BL6 mice (2017) (127) - 22 goats (2012) (128) - 25 isogenic Wistar rats (2010) (129) - 30 number of rabbits (2006) (130)	Animal Study
–	8 participants (2012–2014) ClinicalTrials.gov Identifier: NCT01532076 (105)	10 participants (2015–2018) ClinicalTrials.gov Identifier: NCT02566655 (105)	Clinical Trial

## Conclusion and Future Directions

The burden of osteoporosis is raised by an increase in the proportion of older persons in societies. Routine treatments only alleviate the symptoms partially. Hence, they are not sufficient enough. Therein, regenerative medicine sheds light on the treatment of osteoporosis. Specifically, MSCs therapy is the most common technique of regenerative medicine in osteoporosis treatment. Moreover, using small molecules (e.g., PTH and oxytocin) which employ endogenous stem cells for osteoporosis treatment will be intertwined in future management (20, 144). Despite the many investigations in cell therapy for osteoporosis, further studies are still demanded to fulfill the gaps including the definite differentiation fate and biodistribution of transplanted stem cells. On the other hand, in accordance with growing advances in osteoporosis personalized medicine (the applying of specific medical treatment based on the individual characteristics of each patient), it is required to identify the important bone loss signaling pathways and genes involved in each individual (145–148). In this context, metabolomics evaluation (the principled investigation of small molecules profile in a biological system) (149, 150) also can be helpful to the osteoporosis diagnosis of individuals with a genetic capacity (151, 152). Additionally, the biomedical using of exosomal based treatments will present novel approaches in clinical practice for osteoporosis (116).

## Author Contributions

BA contributed substantially to the conception and design of the study. MP conducted search strategy and data collection. MS and SA-M drafted critical revision of the article. PG and KG revised the article critically for important intellectual content. NM gave final approval of the version to be submitted and any revised version. BL agreed to be accountable for all aspects of the work in ensuring that questions related to the accuracy or integrity of any part of the work are appropriately investigated and resolved. All authors contributed to the article and approved the submitted version.

## Conflict of Interest

The authors declare that the research was conducted in the absence of any commercial or financial relationships that could be construed as a potential conflict of interest.

## Abbreviations

BMD: Bone Mineral Density
WHO: World Health Organization
MSCs: Mesenchymal Stem Cells
Runx2: Runt-related transcription factor 2
OSX: Osterix
Atf4: Activating transcription factor 4
AP-1: Activator Protein 1
FGFs: Fibroblast Growth Factors
TGF-β: Transforming Growth Factor β
IGF-1: Insulin-like Growth Factor 1
BMP: Bone Morphogenetic Protein
PTH: Parathyroid hormone
MMP-9: Matrix Metallopeptidase 9
M-CSF: Monocyte/Macrophage Colony-Stimulating Factor
OPG: Osteoprotegerin
HSC: Hematopoietic Stem Cells
IL-1: Interleukin1
Il-6: Interleukin 6
TNFα: Tumor Necrosis Factor α
DXA: Dual X-ray Absorptiometry
SERMs: Selective Estrogen Receptor Modulators
IV: Intravenous
HRT: Hormone Replacement Therapy
VEGF: Vascular Endothelial Growth Factor
HGF: Hepatocyte Growth Factor
ESCs: Embryonic Stem Cells
iPSCs: induced Pluripotent Stem Cells
VELs: Very small Embryonic-Like stem cells
BM- MSCs: Bone Marrow Mesenchymal Stem Cells.
